# Design, Synthesis and Hydrolytic Behavior of Mutual Prodrugs of NSAIDs with Gabapentin Using Glycol Spacers

**DOI:** 10.3390/ph5101080

**Published:** 2012-10-12

**Authors:** Monther Faisal Mahdi, Hiba Najeh Alsaad

**Affiliations:** 1 Department of Pharmaceutical Chemistry, College of Pharmacy, University of Mustansiriyah, Baghdad, 10052, Iraq; 2 Department of Pharmaceutical Chemistry, College of Pharmacy, University of Basrah, Basrah, 61001, Iraq

**Keywords:** NSAIDs, gastric irritation, mutual prodrug, gabapentin, HPLC

## Abstract

The free –COOH present in NSAIDs is thought to be responsible for the GI irritation associated with all traditional NSAIDs. Exploitation of mutual prodrugs is an approach wherein the NSAID is covalently bounded to a second pharmacologically active carrier/drug with the ultimate aim of reducing the gastric irritation. In this study some NSAIDs were conjugated with gabapentin via ester bonds using glycol spacers with the expectation of reducing gastric adverse effects and obtaining synergistic analgesic effects. The kinetics of ester hydrolysis were studied in two different non enzymatic buffer solutions at pH 1.2 and 7.4, as well as in 80% human plasma using HPLC with chloroform -methanol as mobile phase. Compounds **9a–c** with ethylene glycol spacers showed significant stability at buffer solutions with half lives ranging from about 8–25 h, while the underwent a reasonable plasma hydrolysis (49%–88%) in 2 h. Compound **9d** with a propylene glycol spacer shows a higher rate of enzymatic hydrolysis than the corresponding ethylene glycol compound **9c**. The result of compounds **9a-c** indicate that these compounds may be stable during their passage through the GIT until reaching the blood circulation.

## 1. Introduction

**Figure 1:**
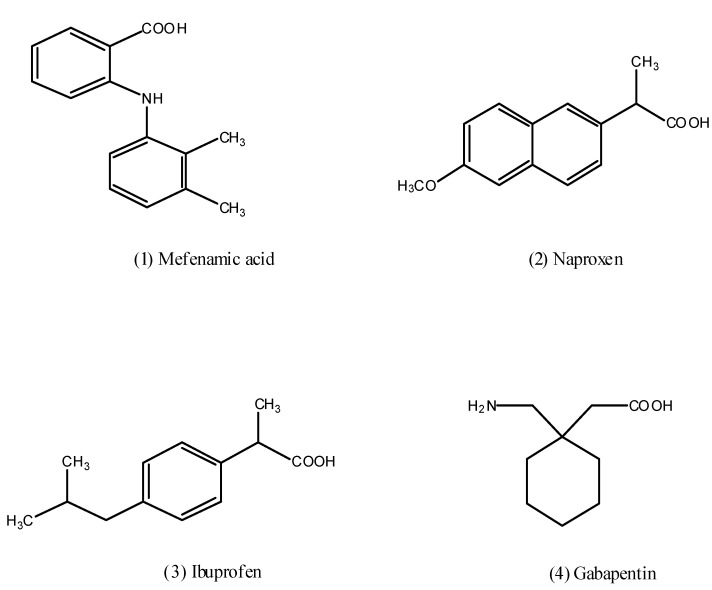
Chemical structures of some NSAIDs and gabapentin.

Non steroid anti-inflammatory drugs (NSAIDs) such as mefenamic acid (**1**), naproxen (**2**) and ibuprofen (**3**) that are represented in Figure 1 constitute a group of heterogeneous molecules that account for a large share of the drug market [1]. These compounds possess one or more anti-inflammatory properties such as analgesic, anti-pyretic, and edema-reducing effect, so they are used for the long and short term management of various conditions, include osteoarthritis, rheumatoid arthritis [2], and musculoskeletal pain [3]. Recent years, epidemiological studies have indicated that NSAIDs are neuroprotective [4], so prolonged use reduces the risk of Alzheimer’s [5]. Clinical studies have provided evidence that NSAIDs are also promising anticancer drugs [6]. However, the relationship between oral intake of NSAIDs and gastrointestinal (GI) side effects like gastric irritation, ulceration, bleeding, and in some cases life threatening conditions, restrict their clinical usefulness [7], so this is the impetus for the development of effective NSAIDs with more favorable GI safety profiles.

Pain, being one of most uncomfortable sensations, represents a protective function warning the body of potentially damaging stimuli [8]. Clinically different types of pain have been identified such as inflammatory pain, that refers to the pain occurring in response to tissue injury and accompanied by a neurogenic inflammation. It results from the release of sensitizing inflammatory substances. The changes following inflammation are generally all reversible, and the sensitivity of the system will be restored when the inflammation has disappeared [9]. A classical example of such pain is that caused by arthritis [10]. Another type of pain is neuropathic pain which is defined as pain arising as a direct consequence of a lesion or disease affecting the somatosensory system [11,12].

Gabapentin (**4**) is an anticonvulsant drug that was synthesized as a structural analog of the neurotransmitter γ-aminobutyric acid (GABA) [13], it is used for treatment of partial seizures with or without generalization [14], as well as its efficacy in the management of chronic pain syndromes, especially neuropathic pain [15].

Mutual prodrugs consist of two drugs chemically linked together, so each therapeutic agent acts as a promoiety to the other [16]. The active moiety selected may have the same biological action as that of the parent drug and thus may give a synergistic action [17], or this moiety may have some additional biological action lacking in the parent drug, thus ensuring some additional benefit [18]. The active moiety may also be a drug that might help to target the parent drug to a specific site or organ or may be use to overcome some side effects of the parent drugs as well [19]. Thus, the mutual prodrug approach is of a great interest, because combination therapy is used for the management of many diseases where therapeutic agents can be co-administered in separate dosage forms, however, there are potential advantages in delivering co-administered agents as a single chemical entity [20].

In view of this background, mutual prodrugs were synthesized by conjugating different types of NSAIDs with gabapentin using glycol spacers in order to ameliorate the NSAID’s gastric irritation by esterification of the free carboxyl group, and produce a synergistic analgesic effect from use two analgesic drugs. In addition gabapentin has been used in the treatment of neuropathic pain that may be associated with different types of inflammation so coupling with different types of NSAIDs will have an additional benefit for treatment of neuropathic pain with enhanced patient compliance from use of a single chemical entity.

## 2. Results and Discussion

### 2.1. Chemistry

N-Protected gabapentin **6** was obtained through reaction of gabapentin (**4**) with *p*-methoxy-benzaldehyde in the presence of an acid as catalyst, as shown inScheme 1.

**Scheme 1:**
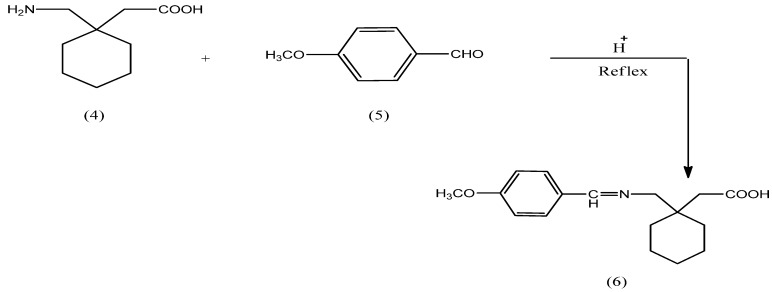
Synthesis pathway of N-protected gabapentin **6**.

The IR spectrum of compound 6 revealed the presence of a strong band at 1,643 cm^−1^ assignable to the imine group, in addition to presence of aromatic (C=C) at 1,585 and 1,500 cm^−1^.

**Scheme 2:**
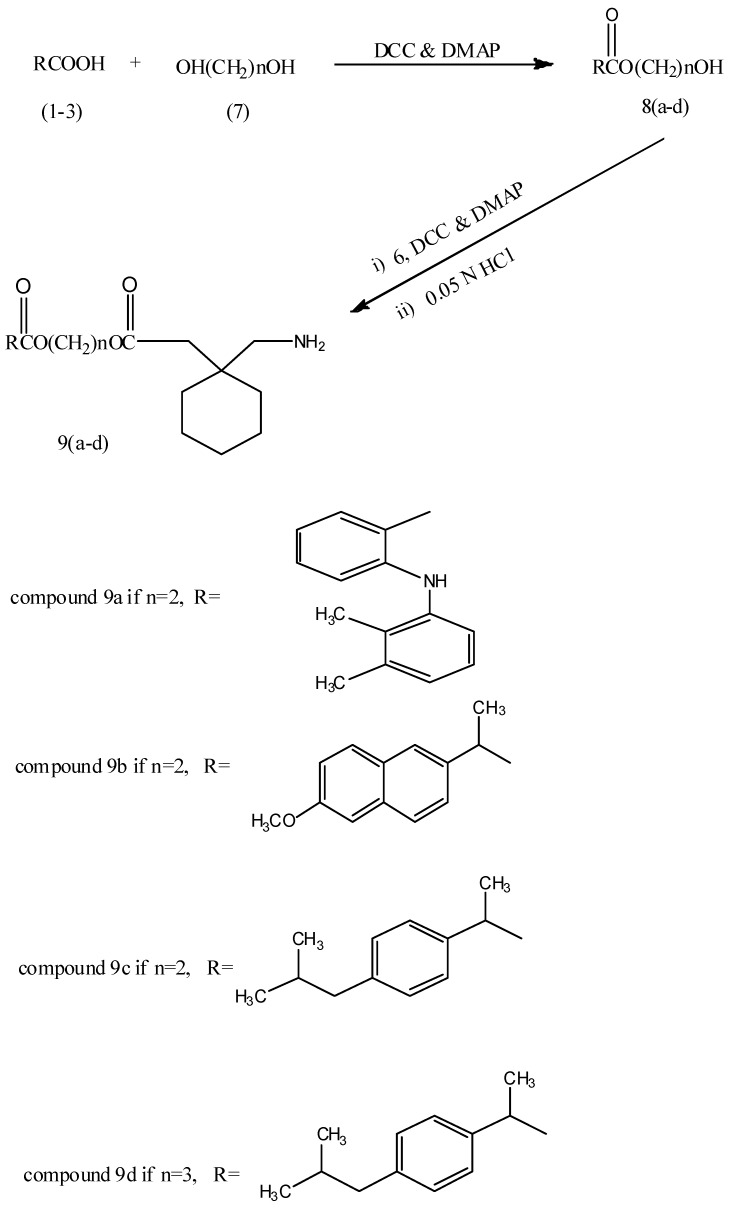
Synthetic pathway of compounds **9a–d**.

The reaction of mefenamic acid (**1**), naproxen (**2**) and ibuprofen (**3**) with ethylene or propylene glycols 7 in the presence of DCC/DMAP afforded the corresponding NSAIDs ester derivatives **8a–d**. The latter were reacted under same reaction conditions with N-protected gabapentin **6** to obtain the final compounds **9a–d** as shown in Scheme 2.

### 2.2. Hydrolysis Studies

The hydrolysis studies of the final compounds were carried out in aqueous buffer solution at different pH values and in human plasma to evaluate their fate during passage through the GIT and after absorption and reaching the blood circulation.

#### 2.2.1. Chemical Hydrolysis

**Figure 2:**
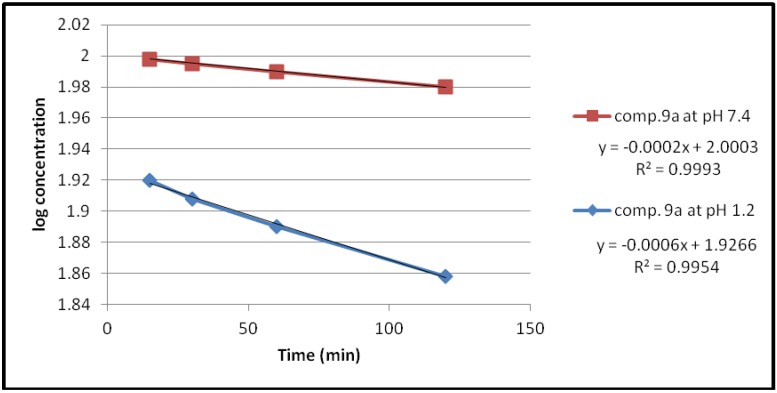
The hydrolysis rate of compound **9a** at different pH values.

**Figure 3:**
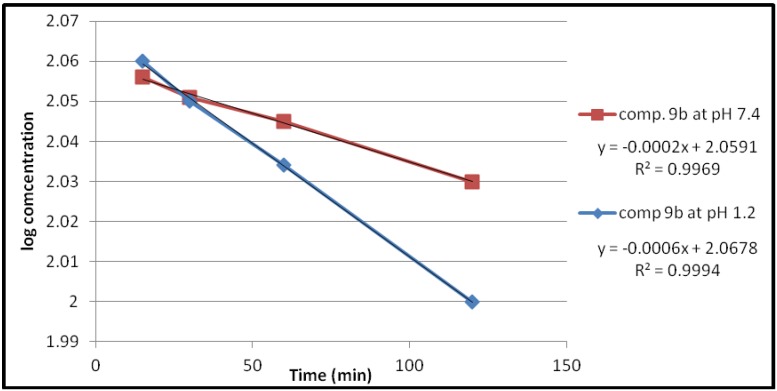
The hydrolysis rate of compound **9b** at different pH values.

**Figure 4:**
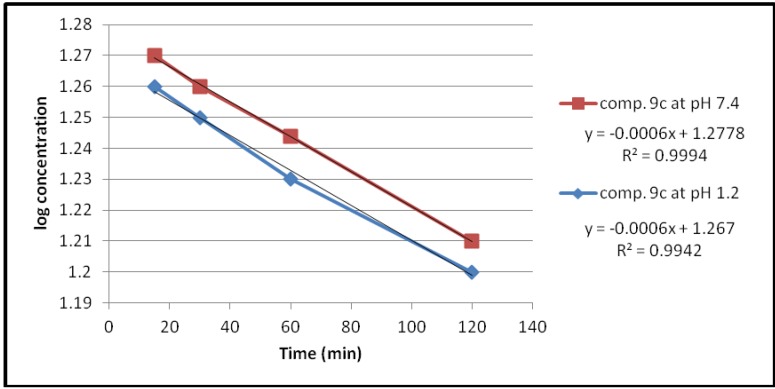
The hydrolysis rate of compound **9c** at different pH values.

**Figure 5:**
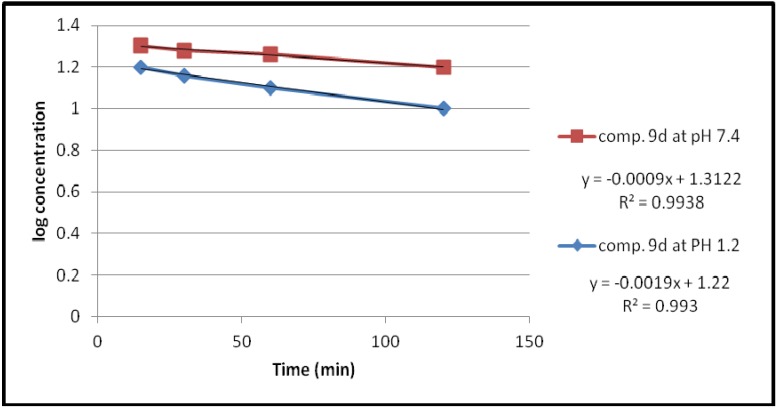
The hydrolysis rate of compound **9d** at different pH values.

Under the experimental conditions used, the hydrolysis of these compounds in aqueous HCl (pH 1.2) and phosphate buffer solution (pH 7.4) at 37 °C followed first order kinetics, since a straight line was obtained from plotting log concentration of the residual products *versus* time as shown in Figure 2, Figure 3, Figure 4, Figure 5.

The observed rate constant of hydrolysis (K_obs_) was calculated from the slope of the curve, and the half life was calculated according to the following equation that derivative from the first order kinetic law [21]:

*t_1/2_* = 0.693/*K_obs_*

The data of chemical hydrolysis that given in Table 1 revealed that these compounds were chemically stable with half lives ranging from about 8 h to 25 h for the ethylene glycol spacer and susceptible to hydrolysis for the propylene glycol spacer for the same compound.

**Table 1:** Kinetic data for the chemical hydrolysis of the compounds (**9a–d**).

Compound	pH	K_obs _(min^−1^)	t_1/2_(min)
**9a**	1.2	1.3818 × 10^−3^	501.51
	7.4	0.4606 × 10^−3^	1504.55
**9b**	1.2	1.3818 × 10^−3^	501.51
	7.4	0.4606 × 10^−3^	1504.55
**9c**	1.2	1.38 × 10^−3^	501.17
	7.4	1.38 × 10^−3^	501.17
**9d**	1.2	4.3757 × 10^−3^	158.37
	7.4	2.07 × 10^−3^	334.78

#### 2.2.2. Enzymatic Hydrolysis

**Figure 6:**
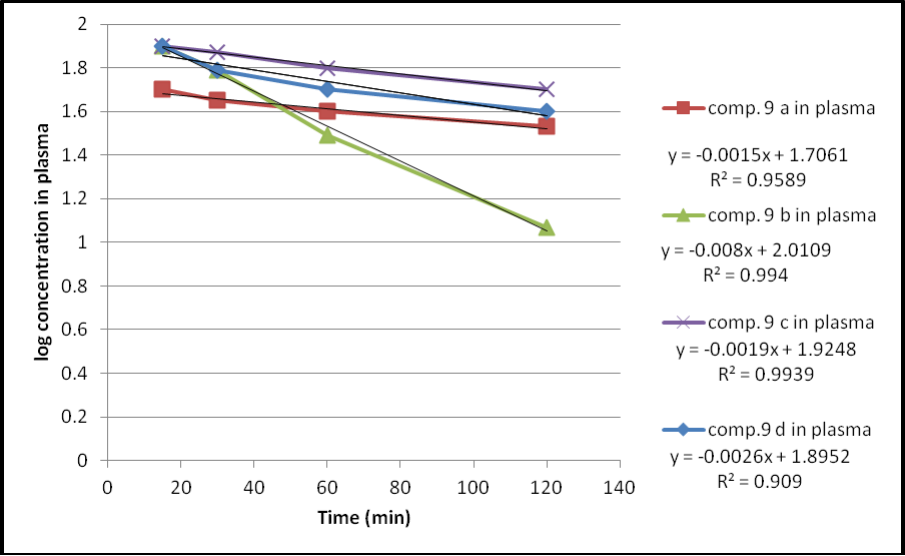
The hydrolysis rate of the compounds **9a–d** in 80% human plasma.

Hydrolysis of these compounds in human plasma also followed first order kinetics as shown in Figure 6.

The plasma hydrolysis data given in Table 2, revealed that these compounds are susceptible to enzymatic hydrolysis with about 38% to 69% hydrolysis occurring during the first hour. Moreover the propylene glycol spacer shows a higher percent hydrolysis than the ethylene glycol one for the same compound. 

**Table 2:** The kinetic data for the plasma hydrolysis of compounds **9a–d**.

Compound	Hydrolysis %				K_obs_	T_1/2_(min)
	15 min	30 min	60 min	120 min		
***9a***	39	57.9	64	66	3.4545 × 10^−3^	200
***9b***	1	37	68.5	88	18.424 × 10^−3^	37.6
***9c***	19	21.4	37.8	49	4.3757 × 10^−3^	158.3
***9d***	13	37	47	59	5.9878 × 10^−3^	115.7

An essential criteria for the prodrugs to be used, is that they should have a good chemical stability [22] and readily undergo enzymatic hydrolysis to release the parent drug [23]. Accordingly compounds **9a–c** would be to be chemically stable during their passage through the gastrointestinal tract. However, compound **9d** with a propylene glycol spacer shows susceptibility to both chemical and enzymatic hydrolysis. 

## 3. Experimental

### 3.1. General

All reagents and anhydrous solvents were used as received from the commercial suppliers (Merck, Darmstadt, Germany, Sigma-Aldrich, Munich, Germany, BDH, Pool Dorset, England and Fluka, Newport News, USA). Mefenamic acid, naproxen and ibuprofen were supplied by the SDI Company (Samarra, Iraq). Gabapentin was purchased from Sigma-Aldrich (Shanghai, China). Melting points were determined by the capillary method using Electrothermal IA9000, Essex, UK. Thin layer chromatography (TLC) was run on Silicagel (60) F_254_ (Merck) to check the purity of the products as well as monitoring the progress of reactions. The identification of compounds was done using U.V. detection and chromatograms were eluted with chloroform-methanol (85:15). FT-IR spectra were recorded by using a Shimadzu model (Kyoto, Japan) spectrophotometer on KBr disks. CHN microanalysis was done by using a Euro EA3000 elemental analyzer (Carlo Erba, Milan, Italy). HPLC analysis was done using a KNAUER A63500 analyzer (Berlin, Germany).

### 3.2. Synthesis of N-Protected Gabapentin ***6***

In a 100 mL round bottom flask equipped with a stirrer and reflex condenser, a solution of gabapentin (5.84 mmol) in absolute methanol (40 mL) was added, and then a solution of *p*-methoxy- benzaldehyde (5.84 mmol) in absolute methanol (3 mL) was added dropwise to the first solution. One drop of glacial acetic acid was added to the reaction mixture which was then refluxed for 5 h. After the completion of the reaction, methanol was removed by rotary evaporator, the yellow residue was collected and recrystallized from absolute ethanol to give a yellow crystals of compound **6** [24] (73% yield). M.p. 151–152 °C. R_f_ = 0.89. IR 3000–2600 (O-H) carboxylic acid, 1699 (C=O) of carboxylic acid, 1643 (C=N), 1585 and 1510 (C=C) and 1253 (C-O-C) cm^−1^.

### 3.3. General Procedure for the Synthesis of Glycol Ester Derivatives of NSAIDs ***8a–d***

To a ice-cooled solution of any one of the NSAIDs (mefenamic acid (**1**), naproxen (**2**) or ibuprofen (**3**), 5 mmol) in a mixture of anhydrous dichloromethane (DCM) and tetrahydrofuran (THF) (20 mL, 1:1), dimethylaminopyridine (DMAP, 0.5 mmol) and ethylene or propylene glycol **7** (15 mmol) were added, and to these stirred mixtures dicyclohexylcarbodimide (DCC, 5 mmol) in anhydrous dichloromethane (5 mL) was added dropwise over 10–15 min, the reaction mixture was stirred at 0 °C for 1 h and then kept in the dark overnight at room temperature. The white precipitate of dicyclohexyl urea (DCU) that formed was separated by filtration and the filtrate was concentrated by evaporation, the precipitate redissolved in ethyl acetate (20 mL) and washed with HCI (0.05 N, 20 mL), 5% sodium bicarbonate and water, respectively, and then dried over anhydrous NaSO_4_ [25], the solvent was evaporated and the products were recrystallized from *n*-hexane; ethyl acetate-*n*-hexane and methanol to give compounds **8a–d** respectively. 

*2-hydroxyethyl-2-((2,3-dimethylphenyl)amino)benzoate* (**8a**) white crystals (53%). M.p. 79–80 °C. R_f_ = 0.73. IR 3335(O-H) of primary alcohol, 3070 (C-H aromatic), 1683 (C=O) of conjugated ester, 1579 and 1510 (C=C) cm^−1^.

*2-hydroxtethyl-2-(6-methoxynaphthalen-2-yl)propanoate* (**8b**): white crystals (55%). M.p. 75–77 °C. R_f_ = 0.78. IR 3327 (O-H) of primary alcohol, 1732 (C=O) of ester, 1604, 1504 and 1483 (C=C) cm^−1^.

*2-hydroxyethyl-2-(4-isobutylphenyl)propanoate* (**8c**): white crystals (35%). M.p. 198–200 °C. R_f_ = 0.85. IR 3325 (O-H) of primary alcohol, 1735 (C=O) of ester, 1516 (C=C) cm^−1^.

*3-hydroxtpropyl-2-(4-isobutylphenyl)propanoate* (**8d**): pale-white crystals (38%). M.p. 149–151 °C. R_f_ = 0.7. IR 3294 (O-H) of primary alcohol, 1735 (C=O) of ester, 1512 (C=C) cm^−1^.

### 3.4. General Procedure for the Synthesis of the Final Compounds ***9a–d***

To ice-cold suspensions of derivative **6** (2.5 mmol) in anhydrous DCM and THF (20 mL, 1:1), DMAP (0.25 mmol) the appropriate derivative **8a–d** (2.5 mmol) in anhydrous DCM (10 mL) was added. To this stirred mixture DCC (2.5 mmol) in anhydrous DCM (5 mL) was added over a period of 10–15 min, and then the reaction mixture was worked up as prescribed in Section 3.3. The products **9a** and **9b** were solids and were recrystallized from ethyl acetate-*n*-hexane (5:20), while **9c** and **9d** were semisolid and purified by treatment with diethyl ether. 

*2-(2-(1-(aminomethyl)cyclohexyl)acetoxy)ethyl-2-((2,3-dimethylphenyl)amino)benzoate* (**9a**): white powder (50%). M.p. 157–159 °C. R_f_ = 0.82. IR 3379 and 3309 (N-H) of primary amine, 1693 and 1672 (C=O) of H-bonding and conjugated esters, 1510 (C=C) cm^−1^. CHN calculated (C_26_H_34_N_2_O_4_): C, 71.21; H, 7.81; N, 6.39; found: C, 71.444; H, 7.93; N, 6.491.

*2-(2-(1-(aminomethyl)cyclohexyl)acetoxy)ethyl-2-(6-methoxynaphthalen-2yl)propanoate* (**9b**): white powder (54%). M.p. 87–90 °C. R_f_ = 0.85. IR 3321 and 3286 (N-H) of primary amine, 1732 and 1697 (C=O) of esters, 1653 (N-H) bending cm^−1^. CHN calculated (C_25_H_33_NO_5_): C, 70.23; H, 7.78; N, 3.28; found: C, 70.312; H, 7.922; N, 3.383.

*2-(2-(1-(aminomethyl)cyclohexyl)acetoxy)ethyl-2-(4-isobutylphenyl)propanoate* (**9c**): semi-solid (42%). R_f_ = 0.91. IR 3327 and 3294 (N-H) of primary amine, 1732 and 1699 (C=O) of esters, 1654 (N-H) bending cm^−1^. CHN calculated (C_24_H_37_NO_4_): C, 71.43; H, 9.24; N, 3.47; found: C, 71.622; H, 9.466; N, 3.631.

*3-(2-(1-(aminomethyl)cyclohexyl)acetoxy)propyl-2-(4-isobutylphenyl)propanoate* (**9d**): semi-solid (45%). R_f_ = 0.85. IR 3323 and 3292 (N-H) of primary amine, 1732 and 1699 (C=O) of esters, 1674 (N-H) bending cm^−1^. CHN calculated (C_25_H_39_NO_4_): C, 71.91; H, 9.41; N, 3.35; found: C, 72.053; H, 9.711; N, 3.492.

### 3.5. Chemical Hydrolysis

The chemical hydrolysis rate of the final synthetic compounds **9a–d** was studied at pH 1.2 and pH 7.4 using phosphate buffer solutions at 37 °C. The total buffer concentration was 0.1 M and the ionic strength (µ) of 1.0 was maintained for each buffer by addition of a calculated amount of sodium chloride. The reaction was followed up by change in AUC of each compound at its specific retention time. The reaction was initiated by adding (100 μL) of stock solution (1 mg/mL) of synthetic compounds in methanol to preheated buffer solutions (5 mL) to obtain a final concentration of 0.02 mg/mL. At a regular intervals (15, 30, 60 and 120 min) aliquots (20 μL) of the reaction mixture were withdrawn and analyzed by HPLC.

### 3.6. Enzymatic Hydrolysis

The enzymatic hydrolysis rate of the final synthetic compounds **9a–d** was studied in human plasma diluted to 80% with isotonic phosphate buffer pH 7.4 at 37 °C. The reaction was initiated by adding 100 μL of stock solution of the target compounds to preheated plasma solution (5 mL), the solution was kept in a water bath at 37 °C, and at appropriate intervals (15, 30, 60 and 120 min), aliquots (300 μL) of solution were withdrawn and deproteinized by adding cold methanol (600 μL). After mixing immediately and centrifuging for 5 min at 4,000 rpm, 20 μL of the clear supernatant was withdrawn and analyzed by HPLC [26].

## 4. Conclusions

Preliminary kinetic study for compounds **9a–c** revealed that these compounds were chemically stable at pH 1.2 and pH 7.4 with half lives ranging from about 8–25 h, while they show a good enzymatic hydrolysis rate in 80% diluted plasma, with more than 60% hydrolysis for compounds **9a** and **9b** during the first hour. Compound **9d**, however, shows susceptibility to both chemical and enzymatic hydrolysis, with a higher rate of enzymatic hydrolysis than compound **9c** which may be attributed to the effect of the spacer length on steric hindrance.

## References

[1] Ajmone-Cat M.A., Bernardo A., Greco A., Minghetti L. (2010). Non-steroidal anti-inflammatory drugs and brain inflammation: Effects on microglial functions. Pharmaceuticals.

[2] Uludag M.O., Ergün B.C., Alkan D.A., Ercan N., Özkan G.Y., Banoglu E. (2011). Stable ester and amide conjugates of some NSAIDs as analgesic and anti-inflammatory compounds with improved biological activity. Turk. J. Chem..

[3] James M.S., Clemence E.H. (2010). Strategies to optimize treatment with NSAIDs in patients at risk for gastrointestinal and cardiovascular adverse events. Clin. Ther..

[4] Parto S.K., Robert F.H. (2009). Evidence for neuroprotection by the fenamate NSAID, mefenamic acid. Neurochem. Int..

[5] Mizushima T. (2010). Molecular mechanism for various pharmacological activities of NSAIDS. Pharmaceuticals.

[6] Wittine K., Benci K., Rajic Z., Zorc B., Kralj M., Marjanovic M., Pavelic K., De Clercq E., Andrei G., Snoeck R. (2009). The novel phosphoramidate derivatives of NSAIDs 3-hydroxypropylamides: Synthesis, cytostatic and antiviral activity evaluations. Eur. J. Med. Chem..

[7] Tomisato W., Tsutsumi S., Hoshino T., Hwang H., Mio M., Tsuchiya T., Mizushima T. (2004). Role of direct cytotoxic effects of NSAIDs in the induction of gastric lesions. Biochem. Pharmacol..

[8] Burian M., Geisslinger G. (2005). COX-dependent mechanisms involved in the antinociceptive action of NSAIDs at central and peripheral sites. Pharmacol. Ther..

[9] Jensen T.S., Finnerup N.B. (2009). Neuropathic pain: Peripheral and central mechanisms. Eur. J. of Pain Supplements.

[10] Jensen T.S. (2008). Pathophysiology of pain: From theory to clinical evidence. Eur. J. Pain Suppl..

[11] Sarah M.S., Cristina E.M., Steven P.W., Srinivasa N.R. (2010). Peripheral opioid analgesia for the treatment of neuropathic pain: Gene mutation to virus mediated gene transfer. Eur. J. Pain Suppl..

[12] Finnerup N.B., Sindrup S.H., Jensen T.S. (2010). The evidence for pharmacological treatment of neuropathic pain. Pain.

[13] Bıyık İ., Gülcüler M., Karabiga M., Ergene O., Tayya N. (2009). Efficacy of gabapentin versus diclofenac in the treatment of chest pain and paresthesia in patients with sternotomy. Anadolu Kardiyol. Derg..

[14] Gupta S.K., Mahajan A., Tandon V. (2004). Gabapentin for the treatment of neuropathic pain. Palliat. Med..

[15] Graeme J.S. (2006). The mechanisms of action of gabapentin and pregabalin. Curr. Opin. Pharmacol..

[16] Abdel-Azeem A.Z., Abdel-Hafez A.A., El-Karamany G.S., Farag H.H. (2009). Chlorzoxazone esters of some non-steroidal anti-inflammatory (NSAI) carboxylic acids as mutual prodrugs: Design, synthesis, pharmacological Investigations and docking studies. Bioorg. Med. Chem..

[17] Ohlan S., Nanda S., Pathak D.P., Jagia M. (2011). Mutual prodrugs-A swot analysis. IJPSR.

[18] Bhosle D., Bharambe S., Gairola N., Dhaneshwar S.S. (2006). Mutual prodrug concept: Fundamentals and applications. Indian J. Pharm. Sci..

[19] Amjad M.Q., Meriem M.R. and Bassam (2011). Synthesis, characterization and in vitro hydrolysis of a gemﬁbrozil-nicotinic acid codrug for improvement of lipid profile. Eur. J. Pharm. Sci..

[20] Hamad M.O., Kiptoo P.K., Stinchcomb A.L., Crooks P.A. (2006). Synthesis and hydrolytic behavior of two novel tripartate codrugs of naltrexone and 6β-Naltrexol with hydroxybupropion as potential alcohol abuse and smoking cessation gents. Bioorg. Med. Chem..

[21] Mechael B.A. (2007). Kinetic of product stability. Pharmaceutics the Design and Manufacture of Medicines.

[22] Kalgutkar A.S., Marnett B.A., Crews B.C., Remmel R.P., Marnett L.J. (2000). Ester and amide derivatives of the nonsteroidal anti-inflammatory drug, indomethacin, as selective cyclooxygenase-2 inhibitor. J. Med. Chem..

[23] Bonina F.P., Montenegro L., Caprariis P., Palagiano F., Capasso A., Sorrentino L. (1996). Pharmacokinetic and pharmacodynemic profile of triethylene glycol indomethacin ester as a new oral prodrug. J. Controlled Release.

[24] Nawaz H., Akhter Z., Yameen S., Siddiqi H.M., Mirza B., Rifat A. (2009). Synthesis and biological evaluation of some Schiff base ester of ferrocenyl aniline and simple aniline. J. Organomet. Chem..

[25] Lee B.S., Yoon C.W., Osipov A., Moghavem V., Nwachokor D., Amatya R., Na R., Pantoja J.L., Pham M.D., Black K.L. (2011). Nanoprodrugs of NSAIDs: Preparation and characterization of flufenamic acid nanoprodrugs. J. Drug Deliv..

[26] Wadhwa L.K., Sharma P.D. (1995). Glycolamide ester of 6-methoxy-2-naphthylacetic acid as potential prodrugs-physicochemical properties, chemical stability and enzymatic hydrolysis. Int. J. Pharm..

